# Effect of Diabetes Mellitus on Survival and Complication Rates of Tooth-Supported Fixed Dental Prostheses (FDPs): Long-Term Clinical Evaluation

**DOI:** 10.3390/jcm14165673

**Published:** 2025-08-11

**Authors:** Ali Alenezi

**Affiliations:** Department of Prosthetics Dental Sciences, College of Dentistry, Qassim University, P.O. Box 6700, Burydah 51452, Saudi Arabia; ali.alenezi@qu.edu.sa; Tel.: +966-163800050 (ext. 2039)

**Keywords:** survival rate, diabetes mellitus, fixed dental prosthesis, retrospective clinical study

## Abstract

**Background/Objectives**: Diabetes mellitus (DM) can adversely affect oral health by compromising immune function and promoting chronic inflammation. This effect can significantly impact the outcomes of fixed dental prostheses (FDPs). This study aimed to evaluate the rates of complications in FDPs in diabetes patients. **Methods**: The study investigated various clinical factors, including technical complications and biological complications. The investigation included diabetic patients (test group) and non-diabetic patients (control group), who were evaluated during their follow-up visits. Clinical and radiographic assessments were performed to determine the cumulative survival rate, and life table survival analyses of FDPs in the presence of complications were performed. **Results**: This study evaluated 1125 FDPs (66.1% in women), with 27.1% in diabetics, over a mean of 9.3 ± 7.7 years. The overall complication rates analysis, using the Mann–Whitney U test, showed a significant difference between diabetic and non-diabetic patients (*p* = 0.002). Diabetic patients had higher biological complications (58.4% vs. 51.1%, *p* = 0.03) and more technical complications (7.5% vs. 6.1%, *p* = 0.382). Poor oral hygiene strongly correlated with failure (72.1% vs. 12.9%, *p* < 0.001). Survival analysis revealed a decline in FDP survival probability to 0.23 for diabetics and 0.33 for non-diabetics at 15 years (*p* = 0.012). **Conclusions**: DM may reduce the durability of fixed dental prostheses, with diabetic patients showing noticeably higher rates of technical and biological complications compared to non-diabetics.

## 1. Introduction

Diabetes mellitus (DM) can exert numerous effects on oral health and significantly impact the outcomes of fixed dental prostheses (FDPs) [1,2]. As a metabolic disorder characterized by chronic hyperglycemia resulting from insulin deficiency or resistance, DM creates a challenging oral environment through multiple pathophysiological mechanisms [3,4]. The systemic consequences of prolonged hyperglycemia—including microangiopathy, impaired immune function, and chronic inflammation—directly compromise periodontal health and prosthetic longevity [5,6]. Diabetic patients can exhibit a higher risk of periodontitis due to upregulated pro-inflammatory cytokines and dysfunctional neutrophil activity [7], which accelerates alveolar bone loss and jeopardizes abutment tooth stability [8]. Furthermore, hyperglycemia-induced xerostomia reduces salivary flow and buffering capacity while elevating glucose levels in gingival crevicular fluid [9], creating an ideal environment for cariogenic bacteria like Streptococcus mutans to thrive [10]. This leads to accelerated caries development, particularly at FDP margins, with diabetic patients demonstrating higher rates of secondary caries compared to non-diabetics [11]. The oral manifestations of DM extend to impaired wound healing, reduced collagen synthesis, and increased matrix metalloproteinase activity, all of which negatively affect soft tissue adaptation to prosthetic margins [12]. Prosthodontic complications in diabetic patients follow distinct patterns: biological failures (caries, periodontitis) dominate in early to mid-term (3–5 years), while mechanical complications (cement failure, fractures) become more prevalent in later stages (10+ years) [11,12]. Material selection plays a crucial role, with zirconia prostheses showing significantly better outcomes (a 43.1% failure rate) compared to porcelain-fused-to-metal (PFM) restorations (a 79.8% failure rate), due to their superior marginal fit and biofilm resistance [13,14]. Optimal management of diabetic patients requiring FDPs necessitates a multidisciplinary approach emphasizing glycemic control, thorough oral hygiene maintenance, and prosthodontic designs that minimize biological and technical risks [14].

While DM is recognized as a significant risk factor for complications in FDPs, the long-term impact of DM on prosthetic outcomes remains insufficiently investigated [15]. The existing evidence fails to adequately address critical questions regarding the influence of glycemic control variations, diabetes duration, and diabetes-related systemic complications on FDP longevity. These knowledge gaps significantly impact clinical decision-making, as current guidelines must rely on extrapolations from limited evidence or studies of non-diabetic populations. The absence of standardized, long-term clinical studies represents a critical gap in prosthodontic research.

The aim of this retrospective clinical study was to evaluate the rates of biological and technical complications in FDPs in a long-term evaluation, with particular focus on assessing whether DM significantly influences complication rates. The investigation tested the null hypothesis that no significant differences exist in overall complication rates between diabetic and non-diabetic patients.

## 2. Materials and Methods

This study, approved by Qassim University’s Ethics Committee, employed a comparative design to evaluate complication rates in tooth-supported FDPs. The investigation included clinical and radiographic examinations for diabetic patients (test group) and non-diabetic patients (control group) during their follow-up visits from November 2024 to May 2025. Inclusion criteria required patients to be over 40 years of age, provide signed informed consent, and have fully tooth-supported FDPs with complete abutment coverage. Implant-supported prostheses and removable partial dentures were excluded. All clinical evaluations followed a standardized protocol involving periapical radiographs using the long-cone technique and comprehensive clinical examinations. The latter included visual inspection of prostheses; mobility testing through buccal, lingual, and axial pressure application; and oral hygiene assessment using the Simplified Oral Hygiene Index (OHI-S), which categorized plaque and calculus accumulation as good, fair, or poor. The periodontal problems were not evaluated in this study as a complication [16]. The clinical and radiographic assessments were conducted blindly by two independent clinicians using a pre-designed form to record data following each patient’s examination.

Complications were classified as either technical (framework fractures, veneer fractures, tooth fractures, mobility, abutment loss, or retention loss) or biological (secondary caries or apical lesions). FDPs were deemed “surviving” if no clinical or radiographic complications were detected and “failed” if they exhibited fractures, debonding, caries, or radiographic evidence of failure. The study followed the guidelines of the Strengthening the Reporting of Observational Studies in Epidemiology (STROBE) checklist (Supplementary Table S1) [17].

Statistical analyses included a life-table analysis for cumulative survival rates, the Kruskal–Wallis test for oral hygiene status comparisons, and the Mann–Whitney U test for assessing differences by gender and material type. All analyses were conducted using SPSS version 30 (SPSS Inc., Chicago, IL, USA), with statistical significance set at *p* ≤ 0.05.

## 3. Results

A total of 415 patients were included in this study, with an age range of 40 to 86 years; 57.1% of the participants were women. From these patients, a total of 1125 FDPs were analyzed, with a mean observation period of 9.3 ± 7.7 years (Table 1). Of the FDPs, 381 (33.9%) were placed in male patients and 744 (66.1%) in female patients. Biological complications were reported in 59.6% of male cases and 49.7% of female cases (*p* = 0.002). No significant difference was found between genders regarding technical complications (*p* = 0.486). However, the overall complication rate was significantly higher in males (64.0%) compared to females (54.3%) (*p* = 0.002). Among the participants, 305 FDPs (27.1%) were in diabetic patients, while 820 (72.9%) were in non-diabetics. Statistically significant differences were observed in the incidence of biological (*p* = 0.03) and overall complications (*p* = 0.002) between diabetic and non-diabetic patients. Table 2 and Table 3 show the life-table survival analysis of FDPs in diabetic and non-diabetic patients with regard to the occurrence of complications.

FDPs located in the maxilla accounted for 59.2% (*n* = 666), while 40.8% (*n* = 459) were placed in the mandible. Although the overall complication rate was slightly higher in mandibular FDPs (60.1%) than in maxillary FDPs (55.9%), this difference was not statistically significant (*p* = 0.154). Patients were categorized based on oral hygiene into good (7.6%), fair (38.2%), and poor (54.2%) groups. Biological complications were significantly more frequent in the poor oral hygiene group (72.1%) compared to the fair (34.0%) and good (12.9%) groups (*p* < 0.001). Similar significant trends were noted for technical complications (*p* < 0.001) and overall complication rates (*p* < 0.001). The majority of FDPs were single crowns (55.7%), followed by conventional bridges (36.5%). The highest rates of biological complications were observed in cantilever bridges (73.9%) and splinted crowns (65.1%). Veneers showed the highest rate of technical complications (19.0%), while cantilever bridges had the highest number of total complications (78.3%). The design of the FDP had a statistically significant impact on all complication types (biological: *p* < 0.001; technical: *p* = 0.002; total: *p* < 0.001). The most commonly used material was porcelain-fused-to-metal (PFM) (76.4%), followed by zirconia (15.6%) and full ceramic (8.0%). Zirconia FDPs had the lowest biological complication rate (25.6%) but the highest technical complication rate (21.9%). Full ceramic restorations showed a low technical complication rate (2.2%). The material type had a significant association with biological (*p* < 0.001) and overall complication rates (*p* < 0.001), but not with technical complications (*p* = 0.097).

**Figure 1:**
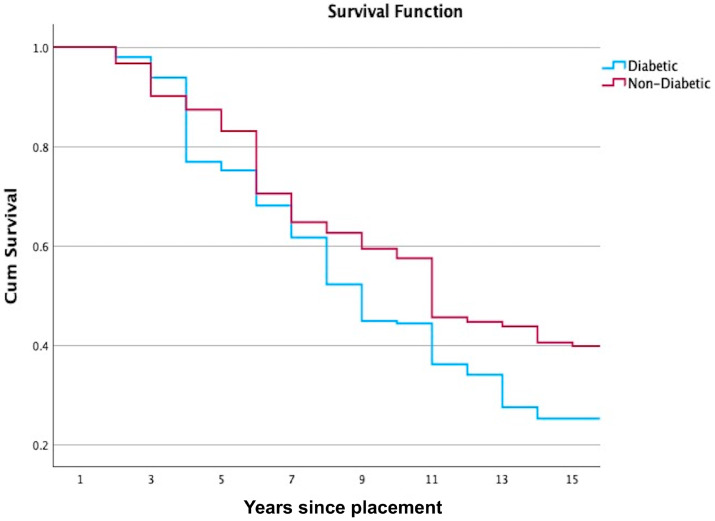
The Kaplan–Meier survival analysis of the occurrence of complications on FDPs between diabetic and non-diabetic patients (*p* = 0.012).

The Kaplan–Meier survival analysis of FDPs in diabetic patients demonstrated a progressive decline in cumulative survival probability from 1.00 at baseline to 0.23 at the 15-year follow-up (Figure 1). For non-diabetic patients, the Kaplan–Meier survival analysis of 820 cases demonstrated a progressive decline in cumulative survival probability from 1.00 at baseline to 0.33 at the 15-year follow-up (*p* = 0.012).

## 4. Discussion

The aim of this study was to evaluate the effect of diabetes on the complication rate of FDPs in the long term. Most studies in the literature have focused on investigating the influence of periodontal issues associated with diabetes [2,5]. There is a dearth of clinical studies that investigate the long-term prognosis and whether diabetes influences the overall complication rate of FDPs. This study found that diabetic patients had similar technical complication rates (7.5% vs. 6.1%, *p* = 0.382) and showed a trend toward increased biological complications (58.4% vs. 51.1%, *p* = 0.03) compared to non-diabetics. Many reports have shown that DM exerts detrimental effects on oral tissues, bone homeostasis, and wound repair processes, which are key determinants of the longevity of FDPs [18,19]. FDPs rely on the structural integrity of supporting dentition, alveolar bone, and periodontal attachments, all of which are undermined by diabetes-associated systemic pathologies including chronic hyperglycemia, immunocompromise, and microangiopathy [7,20]. Furthermore, prosthodontic interventions can potentiate biofilm accumulation on abutment teeth, increasing susceptibility to peri-prosthetic infections and exacerbating metabolic dysregulation in diabetic individuals [21,22].

This study investigated the long-term biological and technical complications associated with tooth-supported FDPs, a widely utilized treatment modality for the restoration of missing or extensively compromised dentition. While the mean survival rate observed in this research (9.3 years) may be considered clinically acceptable, given that most complications were treatable and did not necessarily result in prosthetic failure, the extended average evaluation period of more than nine years surpasses that of comparable studies, enhancing the generalizability of the findings [23,24]. However, a longer observational period would provide a more comprehensive understanding of FDP-related complications. This study exclusively enrolled patients aged 40 years and above to account for the long-term manifestations of DM and to ensure sufficient evaluation time for FDP outcomes.

The assessment of biological and technical complications was conducted through combined clinical and radiographic examinations. Clinical evaluations, involving visual and tactile inspection, are essential for identifying prosthetic integrity issues such as marginal discrepancies or mechanical failures [25,26]. Radiographic analysis, while invaluable for detecting apical pathology, proximal caries, and marginal discrepancies, is limited by the radiopacity of FDP materials, which may obscure caries lesions partially or entirely [26]. This diagnostic challenge is further compounded by clinicians’ variability in the interpretation of radiographic findings, as documented in the literature.

The present study revealed a statistically significant difference in overall complication rates between diabetic and non-diabetic patients during long-term observation (*p* = 0.002). However, a clinically relevant trend emerged for biological complications, including secondary caries and periapical pathology, which occurred more frequently in diabetic patients (*p* = 0.03). While no extant studies have specifically investigated the isolated effects of DM on FDPs, extant literature supports biologically reasonable associations. Pjetursson et al. demonstrated that well-controlled diabetics achieved FDP survival rates comparable to non-diabetics, whereas uncontrolled hyperglycemia was associated with significantly higher failure rates [27]. Complementary findings by Javed and Romanos showed the mechanistic basis for these observations, identifying that hyperglycemia impairs bone metabolism and soft tissue healing, potentially compromising prosthetic longevity [28]. Collectively, these data suggest that while diabetes may not universally affect FDP outcomes, its systemic manifestations, particularly in poorly controlled cases, may elevate risks of biologically driven complications.

The long-term success of FDPs, including crowns and bridges, in diabetic patients is influenced by several critical factors such as glycemic control, periodontal health, prosthetic design, and patient adherence to maintenance protocols [29,30]. This study evaluated the impact of different variables, including gender, oral hygiene status, and prosthesis design/material, on FDP complication rates. The findings demonstrated significant associations between these patient- and prosthesis-related factors and the incidence of complications (Table 1).

This study found that males exhibited numerically higher incidences of biological complications. In contrast, prior research suggests distinct gender-specific failure patterns: females demonstrate higher biological complication rates (e.g., secondary caries), likely due to endocrine-metabolic factors, while males show a predominance of mechanical failures (e.g., abutment screw loosening, framework fractures), attributed to greater occlusal forces and lower treatment adherence [31]. Meanwhile, longitudinal data further revealed gender disparities in 15-year survival rates (males: 29.1% vs. females: 25.7%) [30].

This study identified poor oral hygiene as the most significant predictor of FDP failure. Oral hygiene status was assessed using the OHI-S, a validated measure of plaque and calculus accumulation on tooth surfaces [16,32]. These findings align with those of the existing literature, which reports that diabetic patients with poor oral hygiene have a greater risk of cement failure from biofilm-induced marginal leakage and a fourfold-higher incidence of secondary caries [33,34]. Long-term data further support this association, showing 85% 10-year FDP survival rates with optimal plaque control versus only 60% in neglected cases, with biological complications being the predominant failure mode [35].

This study’s findings reveal that prosthesis design can significantly influence outcomes, with bridges exhibiting failure rates of more than 60% for conventional and cantilever bridge prostheses versus 50.6% and 19% for single crowns and dental veneers, respectively (*p* < 0.001). This corresponds with other studies that have reported a higher incidence of biological and technical complications with bridge prostheses, including caries and loss of retention, compared to single crowns [29,36]. A material-specific analysis revealed significantly lower complication rates for zirconia prostheses compared to full-ceramic and PFM restorations. These findings align with existing evidence demonstrating zirconia’s superior performance, including reduced plaque accumulation [14], attributable to its biofilm-resistant surface properties and decreased subgingival inflammatory response [13,14]. Despite these advantages, PFM prostheses constituted the majority of cases evaluated the most in this study, reflecting their historical dominance as the gold standard for FDPs due to their optimal balance of strength and esthetics. However, contemporary ADA Guidelines (2023) now recommend zirconia materials with supragingival margins and regular HbA1c monitoring for diabetic patients, marking a paradigm shift in prosthetic material selection [37].

The analysis of 1125 FDPs in this study identified biological complications as the primary failure mode, with diabetic patients exhibiting higher rates than non-diabetics. This aligns with the findings of extant literature, which indicates that diabetics face an increased risk of biological complication prevalence, primarily attributed to hyperglycemia-induced xerostomia and impaired neutrophil function [38,39]. Furthermore, different reports have revealed that elevated FDP failure rates in diabetics are due to secondary caries and periodontal breakdown [36,40]. While some studies report conflicting data on the prevalence of caries (e.g., no significant difference in coronal/root caries rates independent of glycemic control) [41], meta-analytic evidence confirms biological failures (caries, periodontitis) as the dominant complication pattern in diabetic patients [36]. Notably, poor glycemic control exacerbates these risks, particularly in older adults with diabetes, who demonstrate significantly higher caries activity and tooth loss [42].

This study identified higher technical complications in diabetic patients, aligning with a systematic review by Sailer et al. [25], who reported elevated bridge fractures in diabetics, attributing this to occlusal overload. Sailer et al. [14] noted higher cement failures in diabetics, while zirconia showed 43% lower debonding than PFM.

While this study did not specifically evaluate periodontal complications, a well-documented concern for diabetic patients with FDPs, existing evidence highlights their critical role in prosthetic failure [15,19]. Diabetic patients exhibit significantly elevated risks of periodontal disease, which compromises abutment tooth stability through pathogen-mediated inflammatory cascades [7,15]. Periodontally compromised patients demonstrate higher FDP failure rates, necessitating rigorous preventive measures: pre-prosthetic scaling and root planing with adjunctive antimicrobials, biomechanically optimized designs (e.g., screw-retained prostheses), and intensive maintenance protocols (three-month recalls with antimicrobial irrigation) [28,37].

The survival rates reported in this study reflect complication-free prostheses rather than absolute treatment failure. Key limitations include the lack of glycemic control data for diabetic participants, which precludes the analysis of how metabolic status influences outcomes. Glycemic control can play an important role in influencing infection risk and wound healing for diabetic patients. Consequently, these findings should be interpreted within the study’s constraints. Future prospective controlled trials incorporating standardized glycemic monitoring may yield more precise survival estimates. The findings of this retrospective investigation are based on the evaluation of FDP treatments performed by different clinicians over various time periods, which may introduce sampling bias. Another limitation is the unequal distribution of FDP types among the study groups, as most evaluated prostheses were PFM. This may not accurately reflect current clinical practice, where PFM restorations are increasingly being replaced by newer materials such as all-ceramic or zirconia-based prostheses.

Despite these limitations, the study identified significantly higher biological complications for fixed prostheses in diabetic patients compared to non-diabetics (*p* = 0.03), suggesting that diabetes may adversely affect prosthetic longevity. These results can inform clinical decision-making when planning restorative treatments for diabetic patients; however, optimal management should also consider the individual’s glycemic control status.

## 5. Conclusions

This long-term study suggests that diabetes may reduce the durability of fixed dental prostheses, with diabetic patients showing noticeably higher rates of biological complications compared to non-diabetics. Overall, the results indicate that diabetes poses substantial challenges to the longevity of dental prosthetics. Prospective controlled studies that monitor HbA1c are needed to validate these findings and to clarify the role of glycemic control. Clinicians should incorporate these evidence-based strategies when planning and maintaining FDPs in diabetic patients to maximize prosthetic survival and patient outcomes.

## Figures and Tables

**Table 1:** Descriptive data of the FDPs included in this study, with follow-up time for the different factors. The statistical unit is the complication rate.

Group		Number of FDPs (%)	Number of FDPs (%) with Sign of Biological Complications	*p*-Value	Number of FDPs (%) with Sign of Technical Complications	*p*-Value	Total Number of FDPs (%) with Complication	(95% CI)	*p*-Value
Gender	Male	381 (33.9%)	227 (59.6%)	0.002	22 (5.8%)	0.486	244 (64.0%)	59–69%	0.002
	Female	744 (66.1%)	370 (49.7%)		51 (6.9%)		404 (54.3%)	51–58%	
Medical condition	Diabetic	305 (27.1%)	178 (58.4%)	0.03	23 (7.5%)	0.382	198 (64.9%)	60–70%	0.002
	Non-diabetic	820 (72.9%)	419 (51.1%)		50 (6.1%)		450 (54.9%)	51–58%	
Location	Maxilla	666 (59.2%)	341 (51.2%)	0.131	45 (6.8%)	0.66	372 (55.9%)	52–60%	0.154
	Mandible	459 (40.8%)	256 (55.8%)		28 (6.1%)		276 (60.1%)	56–65%	
Oral hygiene	Good	85 (7.6%)	11 (12.9%)	<0.001	11 (12.9%)	<0.001	19 (22.4%)	13–31%	<0.001
	Fair	430 (38.2%)	146 (34.0%)		38 (8.8%)		176 (40.9%)	36–46%	
	Poor	610 (54.2%)	440 (72.1%)		24 (3.9%)		453 (74.3%)	71–78%	
FPD design	Veneer	21 (1.9%)	0 (0.0%)	<0.001	4 (19.0%)	0.002	4 (19.0%)	1–37%	<0.001
	Single crown	627 (55.7%)	298 (47.5%)		27 (4.3%)		317 (50.6%)	47–54%	
	Splinted crown	43 (3.8%)	28 (65.1%)		2 (4.7%)		30 (69.8%)	55–84%	
	Cantilever bridge	23 (2.0%)	17 (73.9%)		1 (4.3%)		18 (78.3%)	60–96%	
	Conventional bridge	411 (36.5%)	254 (61.8%)		39 (9.5%)		279 (67.9%)	63–72%	
Type of material	Full ceramic	90 (8.0%)	45 (50.0%)	<0.001	2 (2.2%)	0.097	47 (52.2%)	42–63%	<0.001
	Zirconia	176 (15.6%)	45 (25.6%)		16 (21.9%)		47 (26.7%)	20–33%	
	PFM	859 (76.4%)	507 (59.0%)		55 (6.4%)		554 (64.5%)	61–68%	
Total		1125	597 (53.1%)		73 (6.5%)		648 (57.6%)		

**Table 2:** Life table survival analysis showing the cumulative survival rate for the complications associated with diabetic patients.

Interval Start Time	Number Entering Interval	Number Withdrawing During Interval	Number Exposed to Risk	Number of Terminal Events	Proportion Surviving	Cumulative Proportion Surviving at End of Interval	Std. Error
0	305	0	305	0	1.00	1.00	0.00
1	305	12	299	6	0.98	0.98	0.01
2	287	6	284	12	0.96	0.94	0.01
3	269	40	249	45	0.82	0.77	0.03
4	184	7	181	4	0.98	0.75	0.03
5	173	7	170	16	0.91	0.68	0.03
6	150	3	149	14	0.91	0.62	0.03
7	133	5	131	20	0.85	0.52	0.03
8	108	3	107	15	0.86	0.45	0.03
9	90	1	90	1	0.99	0.44	0.03
10	88	3	87	16	0.82	0.36	0.03
11	69	2	68	4	0.94	0.34	0.03
12	63	1	63	12	0.81	0.27	0.03
13	50	3	49	4	0.92	0.25	0.03
14	43	0	43	0	1.00	0.25	0.03
15	43	0	43	3	0.93	0.23	0.03

**Table 3:** Life table survival analysis showing the cumulative survival rate for the complications associated with non-diabetic patients.

Interval Start Time	Number Entering Interval	Number Withdrawing During Interval	Number Exposed to Risk	Number of Terminal Events	Proportion Surviving	Cumulative Proportion Surviving at End of Interval	Std. Error
0	820	0	820	0	1.00	1.00	0.00
1	820	50	795	26	0.97	0.97	0.01
2	744	49	720	49	0.93	0.90	0.01
3	646	43	625	19	0.97	0.87	0.01
4	584	29	570	28	0.95	0.83	0.01
5	527	38	508	77	0.85	0.71	0.02
6	412	15	405	33	0.92	0.65	0.02
7	364	10	359	12	0.97	0.63	0.02
8	342	15	335	17	0.95	0.59	0.02
9	310	4	308	10	0.97	0.57	0.02
10	296	31	281	58	0.79	0.46	0.02
11	207	6	204	4	0.98	0.45	0.02
12	197	4	195	4	0.98	0.44	0.02
13	189	3	188	14	0.93	0.41	0.02
14	172	2	171	3	0.98	0.40	0.02
15	167	24	155	28	0.82	0.33	0.02

## Data Availability

The raw data supporting the conclusions of this article will be made available by the author on request.

## Supplementary Materials

The following supporting information can be downloaded at: https://www.mdpi.com/article/10.3390/jcm14165673/s1, Table S1: STROBE checklist of items that should be included in reports of observational studies.

## Abbreviations

The following abbreviations are used in this manuscript:

FDPs	Fixed Dental Prostheses
OHI-S	Oral Hygiene Index
DM	Diabetes mellitus
